# Low Fermentation pH Is a Trigger to Alcohol Production, but a Killer to Chain Elongation

**DOI:** 10.3389/fmicb.2016.00702

**Published:** 2016-05-24

**Authors:** Ramon Ganigué, Patricia Sánchez-Paredes, Lluis Bañeras, Jesús Colprim

**Affiliations:** ^1^LEQUIA, Institute of the Environment, University of Girona, Campus de MontiliviGirona, Spain; ^2^Center for Microbial Ecology and Technology (CMET) – FBE – Ghent UniversityGent, Belgium; ^3^Institute of Aquatic Ecology, University of Girona, Campus de MontiliviGirona, Spain

**Keywords:** alcohols, chain elongation, pH, mixed culture, syngas fermentation

## Abstract

Gasification of organic wastes coupled to syngas fermentation allows the recovery of carbon in the form of commodity chemicals, such as carboxylates and biofuels. Acetogenic bacteria ferment syngas to mainly two-carbon compounds, although a few strains can also synthesize four-, and six-carbon molecules. In general, longer carbon chain products have a higher biotechnological (and commercial) value due to their higher energy content and their lower water solubility. However, *de-novo* synthesis of medium-chain products from syngas is quite uncommon in acetogenic bacteria. An alternative to *de-novo* synthesis is bioproduction of short-chain products (C2 and C4), and their subsequent elongation to C4, C6, or C8 through reversed β-oxidation metabolism. This two-step synergistic approach has been successfully applied for the production of up to C8 compounds, although the accumulation of alcohols in these mixed cultures remained below detection limits. The present work investigates the production of higher alcohols from syngas by open mixed cultures (OMC). A syngas-fermenting community was enriched from sludge of an anaerobic digester for a period of 109 days in a lab-scale reactor. At the end of this period, stable production of ethanol and butanol was obtained. C6 compounds were only transiently produced at the beginning of the enrichment phase, during which *Clostridium kluyveri*, a bacterium able to carry out carbon chain elongation, was detected in the community. Further experiments showed pH as a critical parameter to maintain chain elongation activity in the co-culture. Production of C6 compounds was recovered by preventing fermentation pH to decrease below pH 4.5–5. Finally, experiments showed maximal production of C6 compounds (0.8 g/L) and alcohols (1.7 g/L of ethanol, 1.1 g/L of butanol, and 0.6 g/L of hexanol) at pH 4.8. In conclusion, low fermentation pH is critical for the production of alcohols, although detrimental to *C. kluyveri*. Fine control of fermentation pH to final values around 4.8 could allow sustained production of higher alcohols.

## Introduction

The current industrial society depends heavily on fossil resources as a source of energy and raw materials. About 80% of the primary energy used worldwide is obtained from fossil fuels (Sims et al., 2013), while oil is an essential part of almost any material we use within our daily lives. Combustion of fossil fuels is a major source of CO_2_ to the atmosphere (Hartmann et al., 2013), with significant negative impacts on the environment due to air pollution and the enhancement of global warming. In this respect, the depletion of oil reserves and the strategies to mitigate climate change have triggered the development of alternative sources of renewable and carbon-neutral biofuels and chemical building blocks (Marshall et al., 2013).

One of the most promising approaches is the thermal gasification of non-alimentary feedstock sources (i.e., agricultural, forestry, and municipal wastes, biosolids from wastewater treatment plants) to synthesis gas (syngas), a producer gas mainly composed of H_2_, CO, and CO_2_ (Hernández et al., 2013), and its subsequent fermentation to organic acids and/or alcohols. Among its key advantages: (i) its unifying nature allows transformation of heterogeneous resources into a fermentable gas stream using a single technology (Daniell et al., 2012); (ii) biocatalysts are tolerant to impurities such as NO_*x*_, H_2_S, and NH_3_ (Munasinghe and Khanal, 2010; Xu et al., 2011); and (iii) syngas composition may vary considerably due to the heterogeneity of the feedstocks, but biocatalysis is highly independent of CO:CO_2_:H_2_ ratios (Heiskanen et al., 2007).

Biological fermentation of syngas is carried out by acetogens, strict anaerobic bacteria able convert CO_2_ and/or CO to acetyl-CoA through the Wood-Ljungdahl pathway (Ragsdale and Pierce, 2008). Acetyl-CoA can be further transformed to organic acids (i.e., acetate, butyrate), and alcohols (i.e., ethanol, butanol). The latter are of special interest due to their potential as biofuels (Dürre, 2007; Ranjan and Moholkar, 2012). Until now, more than 100 acetogens have been isolated, although not all of them can use CO (or were tested for CO utilization; Diender et al., 2015). Acetic acid is their main end-metabolite, although some acetogenic organisms can produce also ethanol, butyrate, or butanol (Schiel-Bengelsdorf and Dürre, 2012). However, among the plethora of acetogenic bacteria, only two strains, *Clostridium carboxidivorans* P7 and “*Clostridium ragsdalei*” P11, have been reported to produce C6 compounds (caproic acid and/or hexanol) under abundant supply of reducing power (Ramachandriya et al., 2013), during incubation at sub-optimal temperatures (Ramió-Pujol et al., 2015), or using mineral medium and controlling the supply of CO (Phillips et al., 2015).

Syngas fermentation has been mainly studied in pure cultures (Daniell et al., 2012). However, mixed cultures enriched from environmental samples have several potential advantages, such as a higher adaptive capacity (Kleerebezem and van Loosdrecht, 2007), and the selection of the most efficient and effective microbial catalysts (Marshall et al., 2013). Such features confer mixed cultures with a high resistance and resilience to changing environmental conditions, and an increased functional redundancy, aspects that are pivotal to maintain or recover bioreactor productivity in front of many operational eventualities (Marshall et al., 2013). Additionally, positive synergistic relationships can be established within complex bacterial communities. It is then likely that in mixed cultures composed of different bacterial species, the carboxydotrophic production of C2 compounds can be coupled to chain elongation processes, by which short-chain molecules are condensed to produce longer carbon chain products (4, 6, and 8 carbon atoms; Agler et al., 2011; Steinbusch et al., 2011). On the downside, it is challenging to ensure that such communities can deliver a stable production over long periods of time. To date, several works have explored the potential of syngas fermentations in mixed cultures, most of them taking sludge from anaerobic reactors treating organic waste as starting point (Guiot et al., 2011; Alves et al., 2013; Zhang et al., 2013). Among them, Zhang and co-workers demonstrated the feasibility of fermenting a mixture of CO_2_ and H_2_ into short (7.4 g/L acetate; 1.8 g/L butyrate) and medium-chain organic acids (0.98 g/L caproate; 0.42 g/L caprylate) using a mesophilic methanogenic reactor enrichment. Fermentation was carried out in a membrane bio-reactor with pH controlled to 6, thus only organic acids were produced. Besides, Singla et al. (2014) enriched several mixed cultures and optimized their growth conditions for ethanol production, attaining maximum concentrations of 2.3 g/L of ethanol. However, the production of significant concentrations of higher alcohols (i.e., butanol and hexanol) from syngas fermentation by mixed cultures is yet to be reported. This is the objective of the present work, which targets the production of higher alcohols from syngas by mixed cultures, with special attention to how operational conditions affect production dynamics and bacterial community.

## Materials and methods

### Source of inoculum and pre-acclimation to syngas

Inoculum was obtained from the anaerobic digester of the wastewater treatment plant of Girona (Catalonia, Spain). Anaerobic sludge was diluted in a proportion of 1:10 with low-strength phosphate buffered medium and incubated in a serum bottle under stirring at 150 rpm and 37°C. This was flushed with syngas (32% H_2_, 32% CO, 8% CO_2_, and 28% N_2_; Praxair, Spain) to an overpressure of 100 kPa every 2 days, and 80% of the liquid phase was replaced every 7 days with fresh medium to maintain activity of the cultures. After 21 days, once autotrophic acetate production was detected (data not shown), the enriched suspension was inoculated into a controlled bioreactor.

### Media composition

Enrichment of the mixed culture was conducted using a phosphate buffered medium that contained, per liter: 1 g NaCl, 0.25 g NH_4_Cl, 0.05 g Mg(OH)_2_, 0.1 g KCl, and 0.03 g CaCl_2_, 10 mL of vitamin stock solution, 10 mL of trace elements stock solution, 1 g/L KH_2_PO_4_ as a buffer, and 20 mM of 2-bromoethanesulfonate (BES) as a methanogenesis inhibitor (Steinbusch et al., 2009). Vitamin and trace elements stock solutions were prepared according to the ATCC 1754 medium. Medium pH was adjusted to 6.2 with NaOH 40%.

Further reactor experiments with moderate-strength buffered media (25 mM) were conducted using the same medium composition except for the buffers, which were, respectively: (i) phosphate (3.4 g/L KH_2_PO_4_); (ii) 2-(N-morpholino)ethanesulfonic acid (MES; 4.88 g/L); and (iii) bicarbonate (2.1 g/L NaHCO_3_). Additionally, 0.1 g/L of KH_2_PO_4_ was added to the media of MES and bicarbonate experiments to serve as a source of phosphorus.

Media for the solvent production experiment had the same composition as the MES medium above, but was assayed at different MES buffer strengths (0, 10, 25, 50, and 100 mM).

### Reactor set-up

Reactor experiments were carried out in water-jacketed stirred tank reactors with minimum and maximum working volumes of 0.2 and 1 L, and a total vessel volume of 2 L (Afora, Spain). Mixing was provided by a magnetic stirrer at 250 rpm. Temperature was controlled at 37°C by a water bath. Reactors were operated in batch mode. In every batch, cultures were grown during ~20 days, after which 80% of the fermentation broth was replaced with freshly prepared medium. Previous research showed higher productivity of acetogenic strains at initial fermentation pH of 6–7, while low pH-values are required for solvent production (Ganigué et al., 2015). Thus, no external pH control was applied to allow the initial production of organic acids at initial pH around 6, and their subsequent conversion to alcohols at low pH-values. Liquid samples were taken periodically every 2–3 days for the analysis of pH and product composition.

### Enrichment of a syngas fermenting mixed culture

Enrichment of the syngas-fermenting mixed culture was carried out in a 2-L bio-reactor. Syngas (32% H_2_, 32% CO, 8% CO_2_, and 28% N_2_; Praxair, Spain) was injected through a diffusion stone at a flow rate of 2,000 mL/min for a period of 1 min every 4 h. Such high flow rate ensured the complete replacement of the gas headspace with fresh syngas every 4 h (average initial composition: 29.7 ± 2.6% H_2_, 32.4 ± 2.3% CO, 9.8 ± 0.8% CO_2_, and 28.0 ± 1.4% N_2_; *n* = 17). Pressure in the reactor at the beginning of every 4 h cycle was 1.2 ± 0.1 atm.

The reactor was initially started up with 1% v/v of autotrophic inoculum culture (Section Source of Inoculum and Pre-acclimation to Syngas) and was operated in batch mode for 109 days, divided in five batch runs (B1–B5). Samples for bacterial community analysis were taken for each batch. A volume of 15 mL was aseptically collected and centrifuged at 10,000 rpm during 15 min with a centrifuge 5415D (Eppendorf, Germany). Cell pellets were immediately frozen and kept at −20°C until analysis.

### Recovery of chain elongation activity

Three 2-L reactors were used to evaluate how different media buffers (all with initial pHs around 6.1–6.3, but providing different pH profiles throughout the fermentation) affected chain elongation to C6 compounds and the microbial community structure. Reactors were started-up using inoculum from the enrichment reactor (10% v/v) obtained at the end of B5, and were operated in parallel for two consecutive batches. Syngas (32% H_2_, 32% CO, 8% CO_2_, and 28% N_2_;Praxair, Spain) was injected in each reactor through a diffusion stone at a flow rate of 58.3 mL/min for a period of 2 min every 3 h. Such low flows were aimed at decreasing metabolic rates, preventing the fast accumulation of organic acids and the subsequent drop in pH. Samples for bacterial community analysis were taken from each reactor at the end of batch 1 and 2, following the same procedure above-described.

### Consumption of CO and CO_2_/H_2_

The capacity of the microbial community to consume CO and/or CO_2_/H_2_ was assessed in a batch study. Three 500-mL serum bottles containing 90 mL of fresh MES medium were inoculated (10% v/v) with culture from the MES reactor after the completion of the second batch. The three bottles were initially flushed with syngas (32% H_2_, 32% CO, 8% CO_2_, and 28% N_2_; Praxair, Spain) to an overpressure of 180 kPa. Following that, samples for the analysis of gas composition, pH, and culture density (optical density, OD_600_) were drawn periodically.

### Production of higher alcohols using mixed cultures

Batch experiments were performed to further investigate the production of higher alcohols by mixed cultures at different final fermentation pHs. Anaerobic 20 mL glass tubes were filled with 5 mL of mineral media containing different concentrations of MES to dampen the decrease in pH due to the production of organic acids. Five concentrations of MES (0, 10, 25, 50, and 100 mM) were used. A total of 12 tubes were prepared for each MES concentration. All tubes were inoculated using 0.5 mL of mixed culture from the MES reactor after the completion of the second batch. Tubes were thoroughly flushed with syngas (32% H_2_, 32% CO, 8% CO_2_, and 28% N_2_; Praxair, Spain) for 1 min to an overpressure of 200–250 kPa. Following that, three tubes of each treatment were sampled, whereas the remaining nine were incubated at 37°C in a rotary shaker Stuart incubator SI500 (Bibby Scientific Ltd., OSA, UK). Tubes were incubated horizontally to enhance gas–liquid mass transfer. During the batch tests, the headspace of each tube was flushed with syngas twice a day for 1 min to ensure replenishment of gas substrates. The optical density of the tubes was monitored daily to follow the evolution of the experiment. Once all treatments reached the stationary phase, tubes were sampled to determine the final concentration of products, pH, and OD_600_.

### Analytical methods

Concentrations of acetic acid, butyric acid, caproic acid, ethanol, butanol, and hexanol were analyzed using a 7890A gas chromatograph (Agilent, USA) equipped with a DB-FFAP column and a FID detector. Injector and detector were held at 250 and 275°C, respectively. The oven was maintained at 40°C during 1 min, after which temperature increased 5°C/min to 70°C, then 10°C/min to 180°C, and finally 35°C/min to 250°C. This temperature was held during 5 min. Helium was the carrier gas and was injected at 85.11 mL/min. Composition of gas headspace (% vol) was analyzed using a gas chromatograph (Agilent 7890A GC system, Agilent Technologies, Spain) equipped with a fused zeolite capillary column (HP-Molesieve, 30 m × 0.53 mm × 50 μm) and thermal conductivity detector (TCD). The pH of samples was measured using a Basic 20+ pH-meter (Crison, Spain). Optical density (OD_600_) was monitored by measuring the absorbance at 600 nm using a UV-2501(PC) spectrophotometer. Concentration of biomass was determined by a gravimetric test according to standard methods and correlated to OD_600_ based on a calibration curve (APHA, 1995).

### DNA extraction and PCR amplification of bacterial 16S rRNA

DNA was extracted from cell pellets using the FastDNA^®^ SPIN kit for soils (MP, Biomedicals) following the manufacturer's instructions. Partial 16S rRNA gene fragments were obtained by PCR amplification using the bacterial universal primers 357F and 907R. Reaction mixtures and PCR amplification conditions have been described previously (Lane, 1991). A 44 base pair GC clamp sequence was added to the 5′ end of 357F primer for separation of PCR products by DGGE (Muyzer and Smalla, 1998). All chemicals and Taq polymerase used in PCR amplifications were provided by Qiagen (Qiagen Ltd., Sussex, UK). PCR amplifications were performed in a 9700 GeneAmp thermal cycler (Applied Biosystems, Foster City, CA).

### DGGE analysis of 16S rRNA genes

When necessary, different PCR products of the same sample were concentrated and quantified. Twenty-five microliters of concentrated 16S rRNA PCR products (from 400 to 500 ng DNA) were loaded on 6% (v/v) acrylamide-bis-acrylamide gels with a 40–65% urea-formamide denaturing gradient (Bäckman et al., 2003). DGGE was performed in a Ingeny phorU system (INGENY, Netherlands) as described previously (Prat et al., 2009). DGGE gel was run for 17 h at 160 V and stained for 30 min with Sybr^®^ Gold (Molecular Probes Europe, Invitrogen Corporation, UK), for visualization under UV excitation. Images were captured with a GelDoc 2000 system. The more intense bands of every position in the gel were excised using a sterile scalpel. The DNA fragments were recovered by elution in Tris/HCl buffer (10 mM at pH 7.4) at 65°C for 2 h and re-amplified with 357F/907R primer pair.

Digital images of acrylamide gel were analyzed using the GelCompar II v4.0 software (Applied Maths BVBA, Sint-Marteens, Belgium). Original DGGE images are shown as supplementary images (Figures S1, S2), and lanes related to this study, identified. Lanes and bands were manually defined. Band positions in the different lanes were normalized using internal standards within samples.

### Sequencing

Sequencing in forward or reverse direction of 16S rRNA gene fragments was performed by the Macrogen service (Macrogen, Korea). Sequences were examined for the presence of chimeras using the Uchime algorithm (Edgar et al., 2011), manually refined using the Bioedit v7.0 package and aligned using the ClustalW software. Aligned sequences were analyzed with the BLASTn^®^ program at NCBI (http://blast.ncbi.nlm.nih.gov/) and bacterial species identified as closer similarities to known sequences using the nucleotide collection database. Partial 16S rRNA gene sequences were submitted to GenBank public database with accession numbers from KM489062 to KM489069.

## Results and discussion

### Enrichment of a syngas-fermenting mixed culture

**Figure 1:**
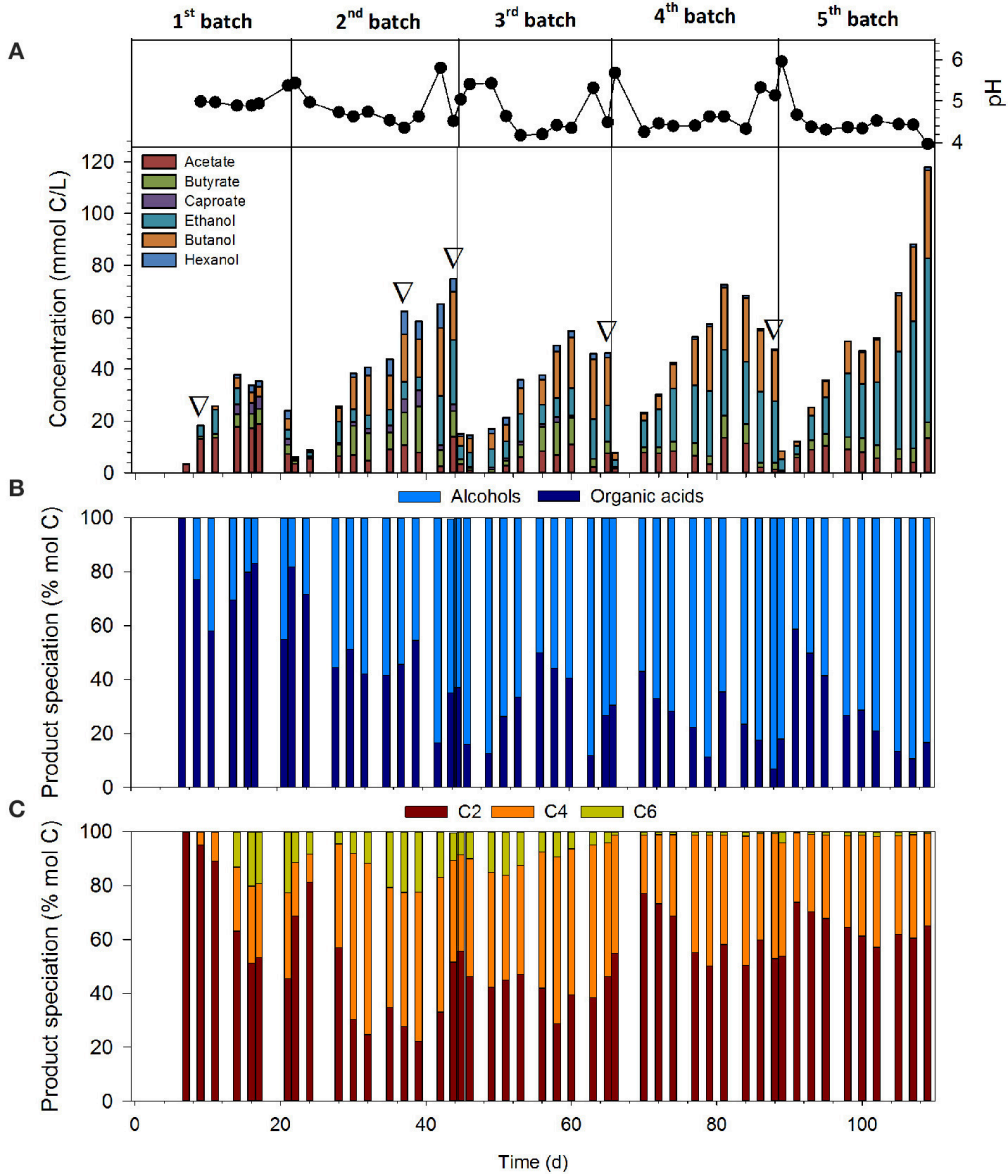
**(A)** Product profile and pH dynamics throughout the enrichment phase. Inverted white triangles indicate the days when samples were obtained for bacterial community analysis; **(B)** percentage of the products in the form of organic acids and alcohols; **(C)** percentage of the products in the form of C2, C4, and C6 compounds.

Figure 1A depicts the total amount of carbon converted into products and pH in the reactor throughout the enrichment phase—Batch 1 (B1) and Batch 2 (B2)—and stable performance—Batch 3 (B3) to Batch 5 (B5)—of the syngas-fermenting mixed culture. Additionally, Figures 1B,C provide further insights on the product speciation in organic acids and alcohols, and the length of product carbon chain.

The total amount of carbon converted into extracellular products increased from B1 to B5 (38 mmol C/L in B1 vs. 118 mmol C/L in B5), indicating an increased performance of the reactor (Figure 1A). Initially, the culture produced mainly acetate (B1), up to concentrations of 20 mmols of carbon as products per liter (mmol C/L). However, as the culture acclimated to the new conditions the reactor reached a stable performance phase, during which product spectrum was dominated by ethanol and butanol. At the end of B3, B4, and B5, the percentage of alcohols represented 83.4 ± 9.8% of the total products, with maximum concentrations of 63 mmol C/L of ethanol and 34 mmol C/L of butanol at the end of B5. Methane production throughout the enrichment phase was negligible (0.09% ± 0.17; *n* = 20), as expected due to BES addition to the medium.

Low extracellular pH has been acknowledged to be a crucial factor triggering solventogenesis, probably due to the increase in the proportion of unionized acid forms at low pH-values (Grethlein et al., 1990; Lewis et al., 2007). In this study, pH was consistently below 4.5 during most part of B3–B5 due to the low buffering capacity of the media. The average concentration of unionized organic acids (encompassing acetic, butyric and caproic acids) during the solventogenic phases of B3–B5 was 2.91 ± 0.84 mmol/L (*n* = 13). Under such conditions, direct production of solvents and/or the re-assimilation of the already produced acids and their subsequent transformation into alcohols are favored over acid production to avoid further inhibition (Jones and Woods, 1986). This is clearly illustrated by periods 70–81 and 93–107 days, during which the concentration of organic acids remained fairly constant while the total concentration of carbon as products increased from 23.4 to 72.6 and 25.2 to 88.3 mmol C/L, respectively, due to the production of alcohols.

During the stable performance phase (B3–B5), about 40% of the carbon fixed as end-products was in the form of four-carbon compounds (C4). This is remarkable considering that C2 compounds are usually the main metabolites derived from syngas fermentation (Bruant et al., 2010; Perez et al., 2013). In addition to that, production of hexanol and caproate was also observed during the transient phase (B1 and B2), although the concentrations of hexanol and caproate, 9 and 6 mmol C/L (0.15 and 0.12 g/L, respectively), were much lower than those previously reported in literature [0.9 g/L hexanol by Phillips et al. (2015), and 0.98 g/L caproate by Zhang et al. (2013)]. However, production of C6 compounds declined from B3, reaching concentrations close to zero at the end of B5. Among all acetogenic bacteria, only *C. carboxidivorans* P7 (Ramachandriya et al., 2013; Phillips et al., 2015; Ramió-Pujol et al., 2015) and “*C. ragsdalei*” P11 (Ramachandriya et al., 2013) have been reported to be capable of directly producing C6 compounds from CO/CO_2_/H_2_. Caproic acid and hexanol production can be also achieved by chain-elongation of 2- and 4-carbon compounds through the reversed β-oxidation pathway, which can be carried out by *Clostridium kluyveri* (Spirito et al., 2014).

**Figure 2:**
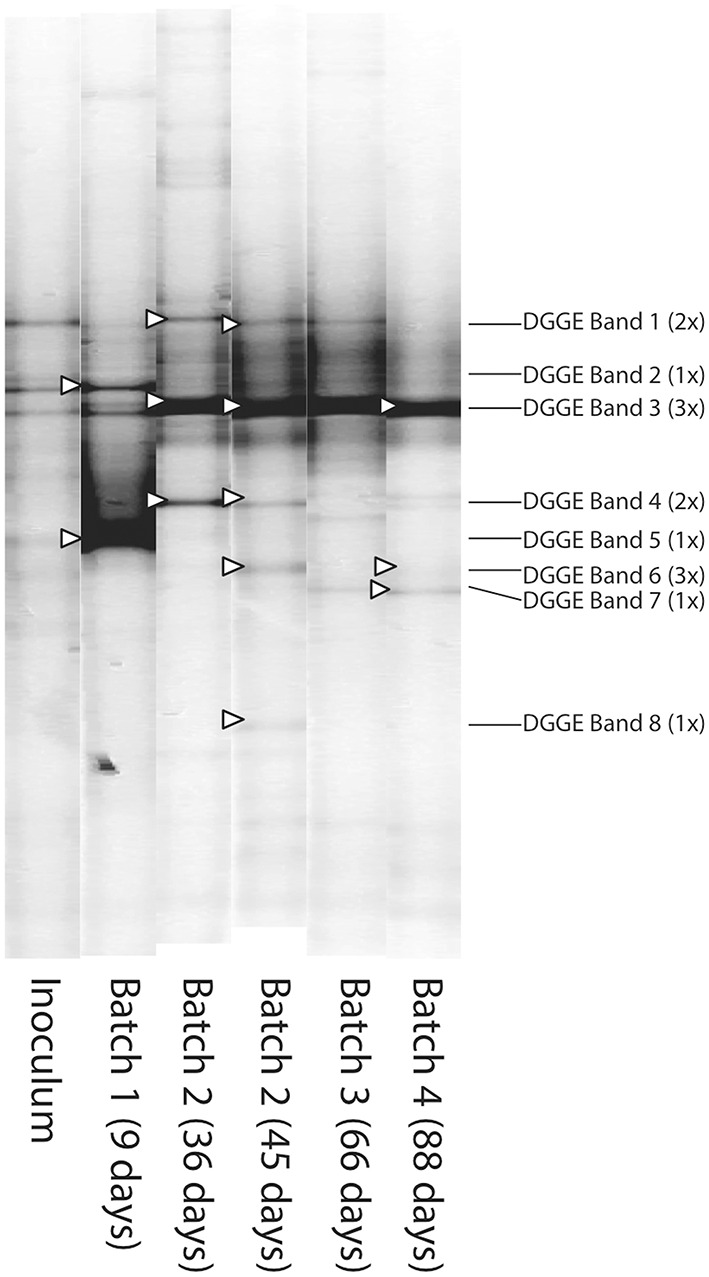
**Negative image composition of SybrGold^®^-stained DGGE gels**. Sampling dates of Batch 1, 2, 3, and 4 are indicated in parenthesis. Lanes are organized on a time-scale basis. Bands indicated with a white arrow head were excised and sequenced. Most probable identification of sequenced bands are indicated at right of the image. Numbers in parenthesis indicate the number of bands excised for every position.

To monitor the evolution of the bacterial community structure, samples were taken from the reactor on days 9, 36, 45, and 88, and analyzed by using PCR-DGGE of the 16S rRNA gene. DGGE profiles (Figure 2) showed that the enriched community was rather simple, with few high intensity DGGE bands per sample. Based on UPGMA algorithm using relative band intensities, fairly homogeneous bacterial communities (> 80% similarity) were found for B2, B3 and B4 samples.

Most probable bacterial species identification corresponding to the most prominent DGGE bands was done by sequencing and comparison to public databases (Table 1). Microbial communities were mainly dominated by bacteria belonging to the phylum *Firmicutes*, which contains most of the identified acetogens and particularly all species related to syngas fermentation (Latif et al., 2014). Only two *Proteobacteria* could be identified, *Acetobacter peroxydans* (DGGE band 6) and *Paludibacterium yongneupense* (DGGE band 8), although they occurred probably as low abundant populations according to band intensities. Precise identifications for DGGE bands 1, 3, and 6, could not be obtained due to more than one exact match to sequences deposited in the NCBI database. Identical 16S rRNA gene sequences have been published for “*C. ragsdalei”* P11, *Clostridium autoethanogenum* and *Clostridium ljungdahlii* (Genbank accession numbers DQ020022, Y18178, and FR733688, respectively). Similar results are obtained with the corresponding 16S rRNA gene sequences of *C. carboxidivorans* P7 and *Clostridium scatologenes* (sequence obtained from DGGE band 1). The presence of the organism detected in DGGE band 4, with a close similarity to *C. kluyveri*, was concomitant to higher chain alcohol and acid production specifically during B2 and to a lesser extent during B3, which supports the hypothesis of the production of C6 compounds through chain elongation of 2- and 4-carbon molecules. DGGE band 4 remained at undetectable levels during B3, B4, and B5, periods in which little or no production of C6 compound was observed. *C. kluyveri* has an optimum growth pH of 7–7.6 (Weimer and Stevenson, 2012; William, 2014), with its activity being seriously compromised at pH below 5 (William, 2014). Therefore, it can be conjectured that the pH below 4.5 during the majority of B3–B5 could be the key factor leading to *C. kluyveri* disappearance from the community.

**Table 1:** **Most probable sequence identification of DGGE bands**.

**Band number**	**Closest bacterial strain**	**Length (bp)**	**Similarity (%)**
DGGE-Band 1 (KM489065)	*Clostridium carboxidivorans* P7	505	100
	*Clostridium scatologenes* K29		
	*Clostridium drakei* FP		
DGGE-Band 2 (KM489066)	*Clostridium aciditolerans* JW-YJLB3	507	98
DGGE-Band 3 (KM489064)	*Clostridium ljungdahlii* DSM13528	543	100
	“Clostridium ragsdalei” P11		
	*Clostridium autoethanogenum* DSM10061		
DGGE-Band 4 (KM489067)	*Clostridium kluyveri* 15	506	100
DGGE-Band 5 (KM489068)	*Acetobacterium fimetarium* DSM8238	498	98
DGGE-Band 6 (KM489062)	*Acetobacter peroxydans* JCM25077	503	99
	*Acetobacter papayae* 1–25		
DGGE-Band 7 (KM489069)	Uncultured *Firmicutes* clone	375	94

*Similarity values and closest match to sequences of cultivated bacteria in GenBank nucleotide collection database are indicated (reference RNA sequences, accession November 2015). Accession numbers appear in parentheses*.

### Recovery of chain elongation activity

**Figure 3:**
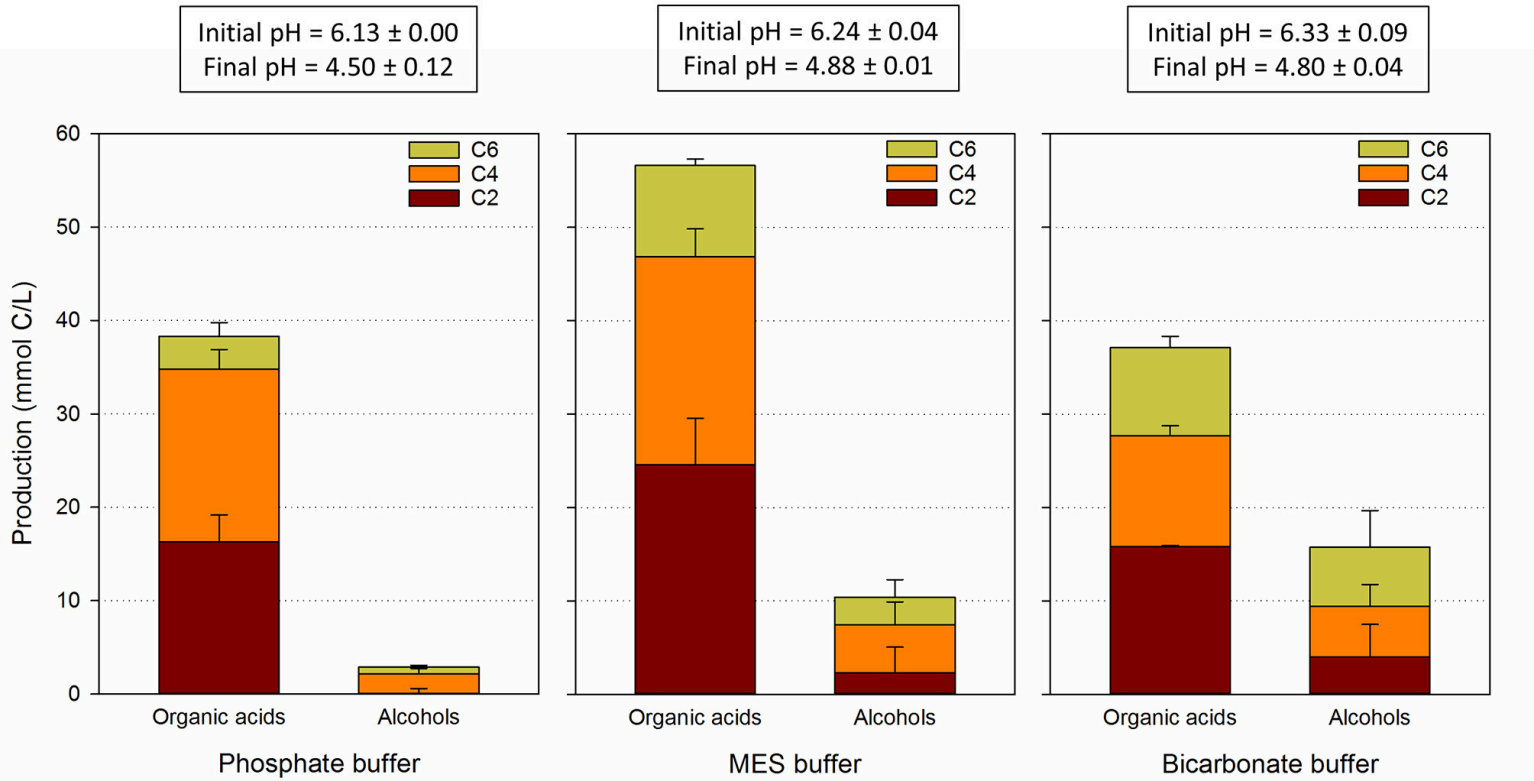
**Net production of C2, C4, and C6 organic acids and alcohols for each media buffer**. Results are an average of two consecutive batch runs.

Three identical reactors, each containing mineral media with different buffering agents were started up using enriched carboxydotrophic inoculum from the previous experiment at the end of B5. The three reactors were operated in parallel for two consecutive batch cycles. The net total production of alcohols and acids (based on the initial and final concentrations) in each media and initial and final pHs (average of two batch runs) are depicted in Figure 3. Further details on the evolution of the concentrations of the fermentation end-products and pH throughout each batch are presented in Supplementary Information (Figures S3–S5).

Overall, the reactor with MES buffer fixed a total of 67.1 ± 9.7 mmol C/L, a concentration significantly higher than those of the experiments with phosphate and bicarbonate buffer (40.5 ± 0.81 and 52.8 ± 9.8 mmol C/L, respectively). Such difference in fixation/production was likely due to the higher buffering capacity of MES over the other two buffers in the pH range 5–6 (Figure S6). The product spectra obtained for each buffer was slightly different. Acetate and butyrate were the main fermentation products in all cases. However, significant production of C6 compounds (caproic acid and hexanol) was detected in both MES and bicarbonate experiments, with total C6 concentrations of 12.8 ± 2.5 and 15.7 ± 5.1 mmol C/L, respectively. Caproic acid was also produced in the phosphate buffer experiments (mainly in the first batch), although in lower amounts, 4.2 ± 1.6 mmol C/L. It is important to note that the use of bicarbonate buffer at pH below 6 may have resulted in an additional supply of CO_2_. However, given the frequent supply of fresh syngas and the high solubility of CO_2_, this should have not affected the outcome of the experiment in any significant way.

**Figure 4:**
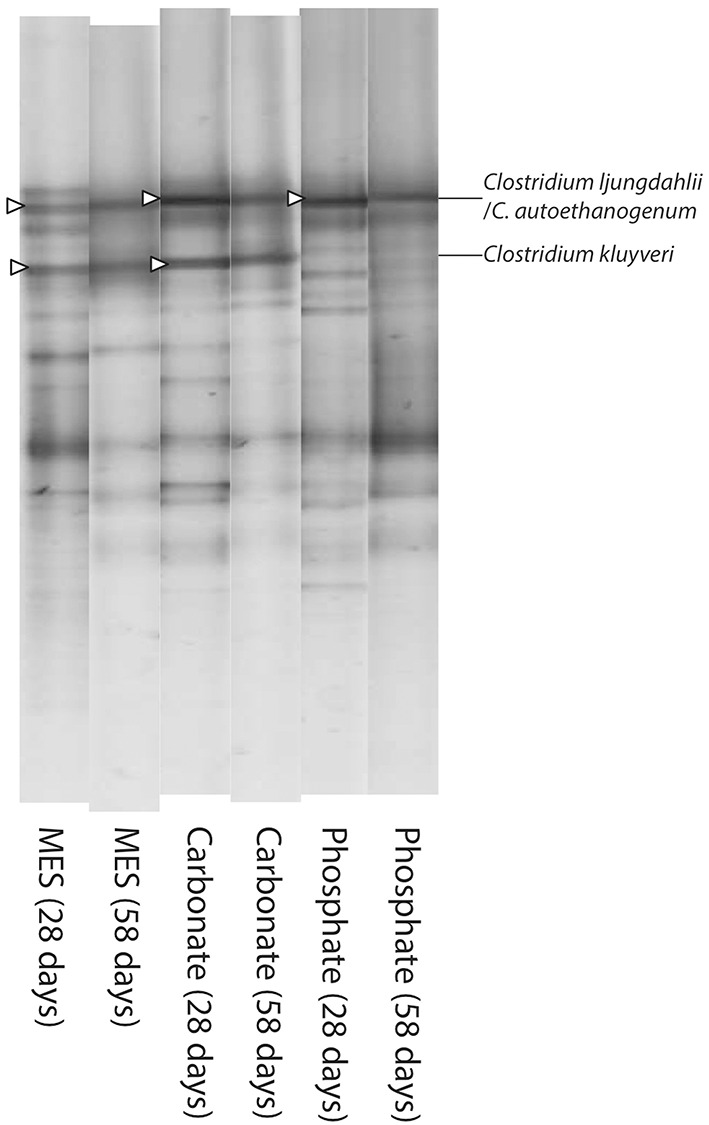
**Negative image composition of SybrGold^®^-stained DGGE gels**. Batch experiments are indicated by their buffer (MES, Carbonate or Phosphate) and the sampling date in parenthesis. Bands indicated with an arrow head were excised and sequenced to confirm identifications reported in Table 1.

At the end of the first and second batch periods, all bacterial communities were dominated by an acetogenic bacterium with identical 16S rRNA sequence to that observed in DGGE band 3 (Figure 4, Table 1) probably being the key organism fixating inorganic carbon into organic products. Gas consumption experiments conducted in 0.5 L-serum bottles showed that the mixed culture was able to grow on both CO and CO_2_/H_2_ (Figure S7). It is thus likely that the abovementioned bacterium was responsible for the consumption of both substrates. Among the three bacterial species showing an identical partial 16S rRNA sequence as the one obtained here, production of C4 and C6 compounds have only been reported for “*C. ragsdalei*” P11. In contrast to the experiment performed here, C4–C6 production by P11 occurred in abundant supply of reducing power (Phillips et al., 2015). More likely, production of both C4 and C6 in our tests can be attributed to a chain elongation mechanism. Although no reliable data on relative abundance of a certain bacterial species can be obtained from intensity measurements of DGGE bands, the presence of DGGE band 4 at high densities coincided with those periods in which higher amounts of C4 and C6 compounds were produced. DGGE band 4 appeared at higher intensities in MES and bicarbonate buffered media, whereas this band remained almost undetectable in the phosphate buffer reactor.

Changes in DGGE band 4 intensities are hypothesized to be linked to fermentation pH. In the MES and bicarbonate buffer experiments, pH was consistently above 5 throughout most of the fermentation period, and only decreased to 4.88 ± 0.01 and 4.80 ± 0.03, respectively, during the final day of the experiment. The final pH of the phosphate buffer experiments was about 0.2 pH units lower, 4.50 ± 0.12, which may help explaining why *C. kluyveri* did not regain a significant role in phosphate buffered media. Finally, one could argue that the concentration of undissociated organic acids could have also played a role in defining prominent population in the reactors and determining product speciation, but no significant differences in the concentration of undissociated organic acids profile were observed across the three reactors (Figure S8).

### Minimum pH for stable production of higher alcohols using mixed cultures

**Figure 5:**
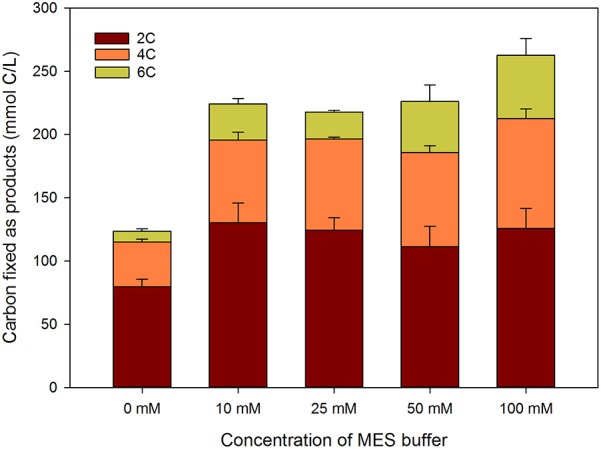
**Overall carbon fixation of the batch fermentation experiments with different MES concentrations (*n* > 6)**.

**Figure 6:**
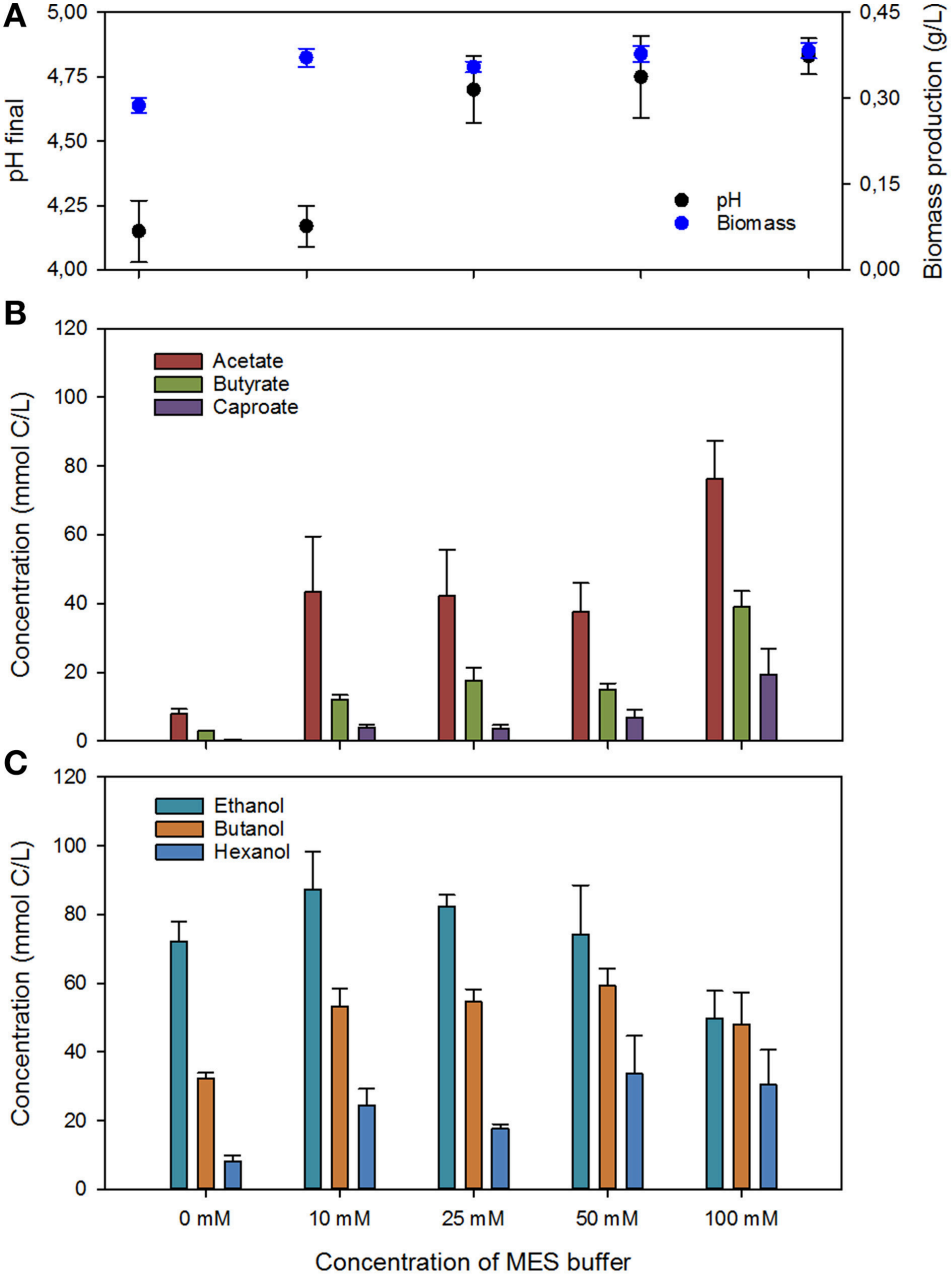
**Results of the batch fermentation in media with different MES concentrations (*n* > 6)**. **(A)** Final pH and concentration of biomass as dry weight; **(B)** net production of organic acids; and **(C)** net production of alcohols.

Batch experiments were performed to further investigate the role of final fermentation pH on product speciation. Several concentrations of MES buffer were used to obtain different buffering capacities and final fermentation pHs. Overall carbon fixation of each test is shown in Figure 5, whereas final concentrations of the different compounds, together with final fermentation pH and biomass are depicted in Figure 6.

Results showed that the better the buffering capacity (and hence the higher the pH during the fermentation), the higher the amount of carbon fixed into products (Figure 5). The batch tests without MES had the lowest productivity, with only 123 ± 15 mmol C/L converted into products. Tests with concentration of MES of 10, 25, and 50 mM had similar carbon fixations (ca. 225 mmol C/L), while the 100 mM MES exhibited the highest carbon fixation (275 mmol C/L).

In terms of product speciation (Figure 6), the highest proportion of alcohols was observed for the unbuffered tests (90.6%), and decreased to around 70% for the experiments at 10, 25, and 50 mM of MES, and further to 45% in case of the 100 mM of MES. Such behavior was likely due to the higher concentrations of buffer providing better pH dampening and delaying the drop in pH and accumulation of high concentration of undissociated organic acids. However, it is important to bear in mind that part of the ethanol could have also been used for chain-elongation, resulting in a decrease in the concentration of ethanol, but an increase in the concentration of C4 and C6 compounds. Production of C2, C4, and C6 compounds was fairly constant in the experiments at 10, 25, 50, and 100 mM of MES, with slightly higher concentrations of C6 compounds being produced in better buffered media. The highest production of C6 compounds (50 mmol C/L) and higher alcohols (59 mmol C/L of butanol and 34 mmol C/L of hexanol) was attained at a final fermentation pH of 4.75. Lower final pH did not increase the concentration of higher alcohols in the fermentation broth, whereas higher final pH of the fermentation test decreased the fraction of C4 and C6 alcohols (i.e., test at 100 mM of MES).

### Implications and future prospects

In this study, a stable carboxydotrophic culture able to grow on both CO and CO_2_/H_2_ was enriched from a sludge sample obtained from an anaerobic digester of a wastewater treatment plant. Given the global distribution of anaerobic digestion, this source of inoculum could be a suitable platform for syngas fermentation to organic acids or bioalcohols. However, anaerobic digestion is applied to the treatment of a plethora of industrial and agricultural wastes, leading to highly heterogeneous microbiomes (Zhang et al., 2014). Future work should study the enrichment of carboxydotrophic bacteria and mixed microbial communities from anaerobic digester sludge from different origins and characteristics.

The present results prove the feasibility of fermenting syngas to higher alcohols using an enriched mixed culture as a biocatalyst. Butanol can be produced from direct syngas fermentation by several well-known strains. However, the production of C6 compounds, is far more challenging, and to date only two strains have been proven to have the capacity to produce both caproic acid and hexanol under specific conditions (Ramachandriya et al., 2013; Phillips et al., 2015; Ramió-Pujol et al., 2015). The proposed approach takes advantage of the synergistic relationship between acetogenic bacteria and chain elongating bacteria to produce C4 and C6 compounds. Concentrations of butanol and hexanol produced by the mixed culture (1.1 g/L of butanol and 0.6 g/L of hexanol) are comparable to those obtained using pure cultures of *C. carboxidivorans*—1.09 g/L of butanol and 0.94 g/L of hexanol, Phillips et al. (2015); 1.07 g/L of butanol and 0.84 g/L of hexanol, Ramió-Pujol et al. (2015)—and show the potential of the proposed approach. Recently, Diender et al. proved that *C. autoethanogenum* and *C. kluyveri* can grow in a co-culture and produce a mixture of medium chain fatty acids and higher alcohols (Diender et al., 2016). The co-culture produced concentrations of butanol and hexanol of 0.42 and 0.41 g/L, respectively.

Care must be taken in selecting adequate operational conditions for mixed cultures (and co-cultures). Among them, pH is probably the most critical, as low pH are needed to trigger solventogenesis, but pH below 4.5–5 pose a serious threat to the viability of chain elongation in the mixed culture. Experimental data of this work suggest that operation at a minimum fermentation pH of 4.7–4.8 should maximize alcohol production and yet allow the growth of the chain-elongating organisms, such as *C. kluyveri*. Diender and co-workers screened the viability of the co-culture in the pH range 4–7, concluding that it was only functional at pH 5.5–6.5. Besides, no significant difference in production was observed within the tested range of viable pH (Diender et al., 2016). Such discrepancy is likely linked to the fact that the mixed culture was composed of different species, with slightly different growth pH range as those of the co-culture. Future work should further investigate the synergistic interactions between the different species of the mixed culture, including their long-term stability and how changes in operational conditions (especially pH) shift the carbon and electron flows.

One of the bottlenecks of syngas fermentation is gas solubility, particularly of H_2_ and CO. Production of higher alcohols relies heavily on the availability of reducing power, and thus production rates and product titers obtained in this study could be substantially improved when using enhanced mass-liquid transfer reactors, such as membrane bio-reactors (Zhang et al., 2013), or systems with cell recirculation (Grethlein et al., 1991; Richter et al., 2013). However, care must be taken that CO is rapidly consumed, as this could be inhibitory to the activity of *C. kluyveri* (Diender et al., 2016). Performance could be also enhanced by using optimized media (Phillips et al., 2015) and operational conditions (Singla et al., 2014). Future research should aim to establish the potential of the mixed culture approach for industrial application, both in terms of production rates, titers and yields, but also on the long-term stability of the culture. Another important aspect that should not be overlooked is the product specificity. In this study, syngas fermentation using mixed cultures yielded a mixture of products which, together with the low titers obtained, would make the product recovery challenging at best. Optimal reactor design and operation could increase product titers, but efforts should also target a more selective production to make easier the downstream processing of the broth. Finally, it is important to bear in mind that higher alcohols are significantly more toxic than ethanol (Lee et al., 2008), and accumulation of higher concentrations of these will unavoidably end up in decreased performance due to activity inhibition unless in-line product extraction is integrated in the processes design.

## Conclusions

- This study proved for the first time the fermentation of syngas to higher alcohols by a mixed microbial culture enriched from natural communities, with maximum concentrations of 1.1 g/L of butanol and 0.6 g/L of hexanol.
- The enrichment process yielded a bacterial community mainly composed of two carboxydotrophic bacteria, i.e., *C. ljungdahli* and *C. carboxidivorans*, and a chain elongating bacteria *C. kluyveri*, on the basis of 16S rRNA fragment gene sequence comparisons.
- Acidic fermentation pH is required to trigger solventogenesis, but fermentation pH around 4.5 are detrimental to *C. kluyveri*. A minimum fermentation pH of 4.7–4.8 should allow production of solvents at a pH compatible for growth of *C. kluyveri*.

## Author contributions

RG performed the experiments and wrote the manuscript. PS performed part of the experiments. LB conducted the community analysis and reviewed and revised the manuscript. JC reviewed and revised the manuscript. All authors gave approval for publication of the manuscript.

### Conflict of interest statement

The authors declare that the research was conducted in the absence of any commercial or financial relationships that could be construed as a potential conflict of interest.
