# Supplementary figures and images for: Low Fermentation pH Is a Trigger to Alcohol Production, but a Killer to Chain Elongation
Source: Front Microbiol. 2016 May 24;7:702. doi: 10.3389/fmicb.2016.00702 (PMC4877396; doi:10.3389/fmicb.2016.00702)

Batch 3 (66 days)

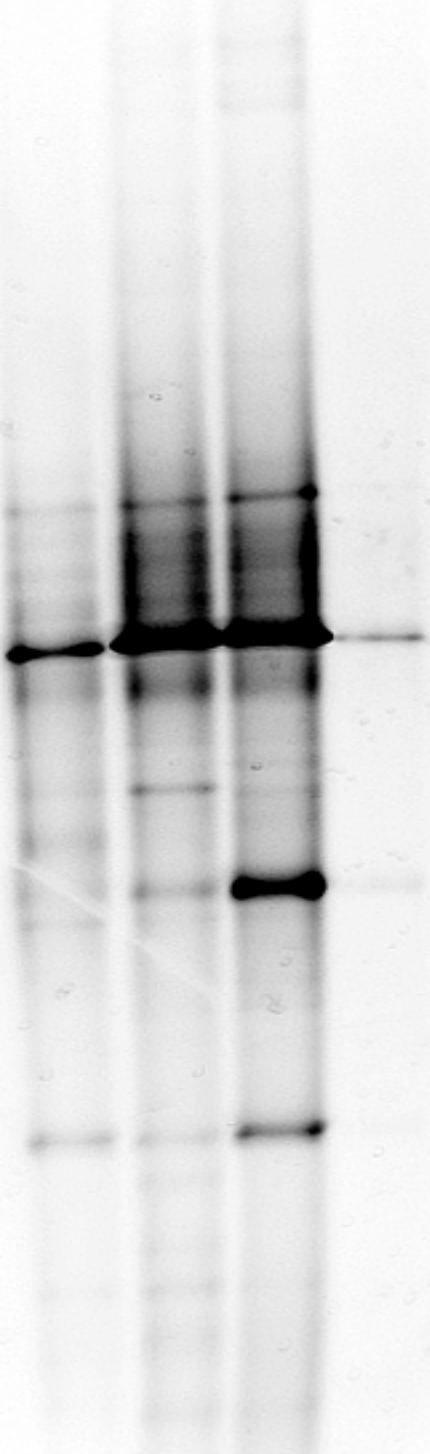

Batch 2 (36 days)

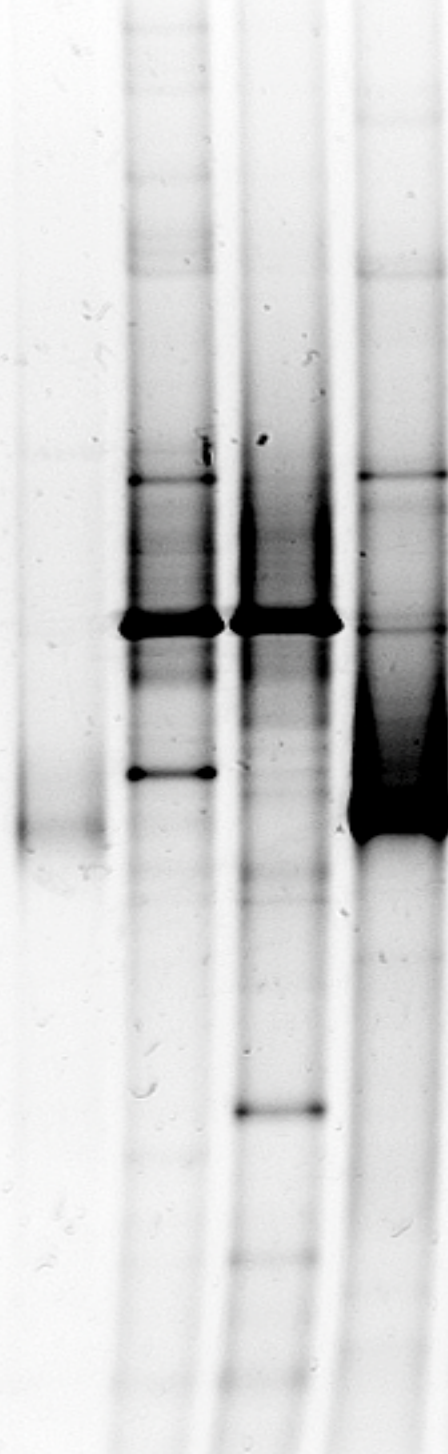

Batch 2 (45 days)

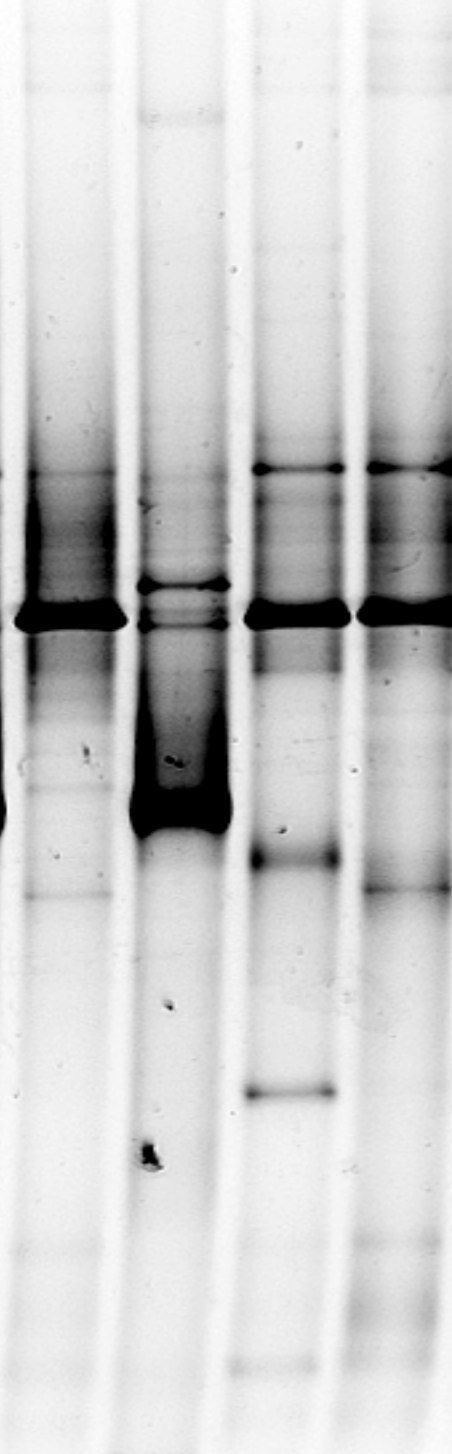

Batch 1 (9 days)

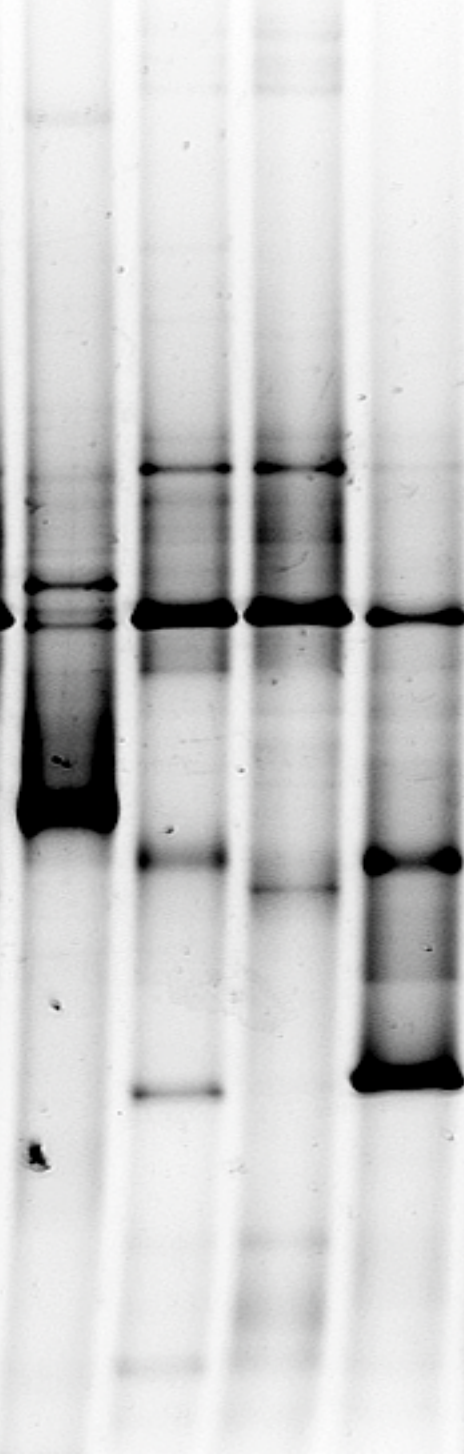

Inoculum

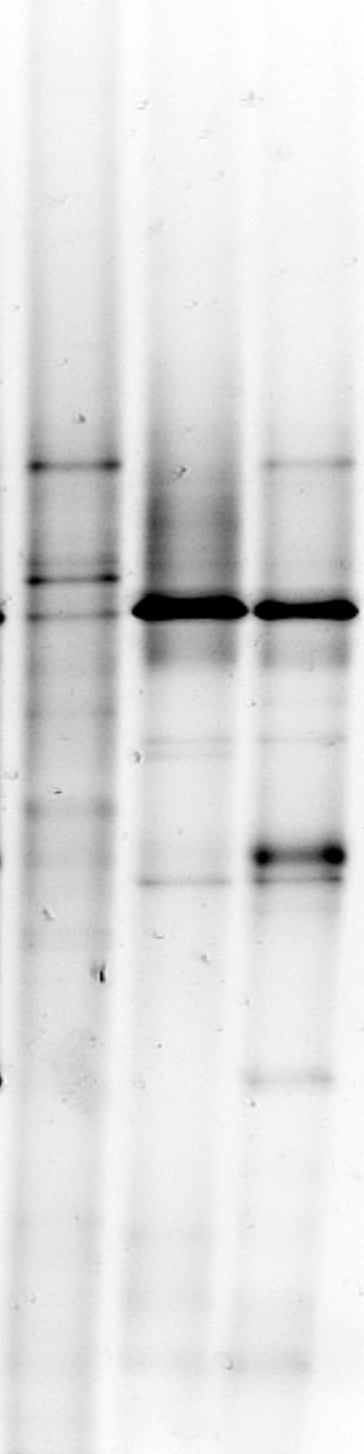

Batch 4 (88 days)

Supplement: Supplementary file 1 [file Image1.PDF]

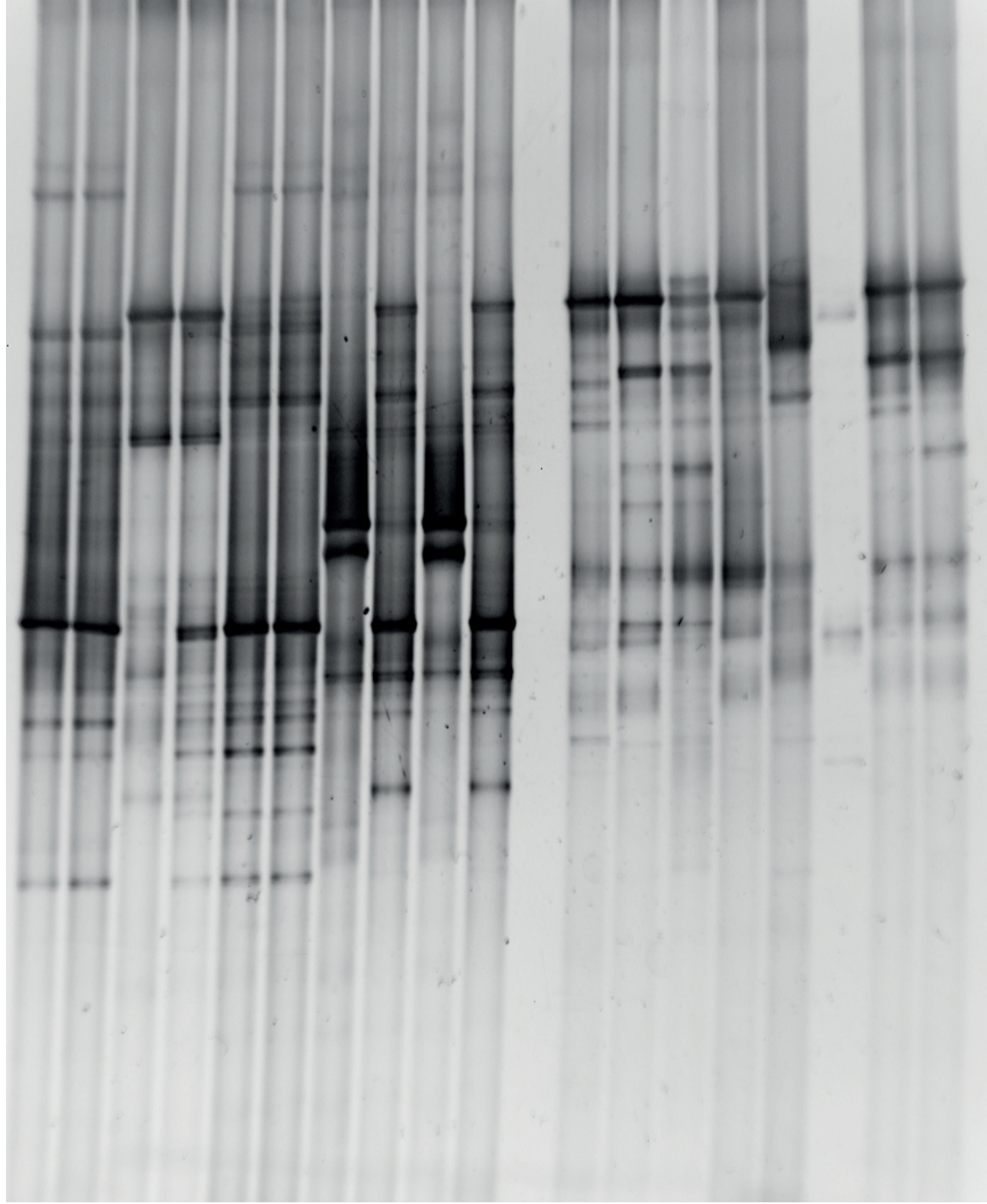

5- Phosphate (28 days)

4- Carbonate (28 days)

3- MES (28 days)

2- Phosphate (58 days)

6- MES (58 days)

7- Carbonate (58 days)

Supplement: Supplementary file 2 [file Image2.PDF]

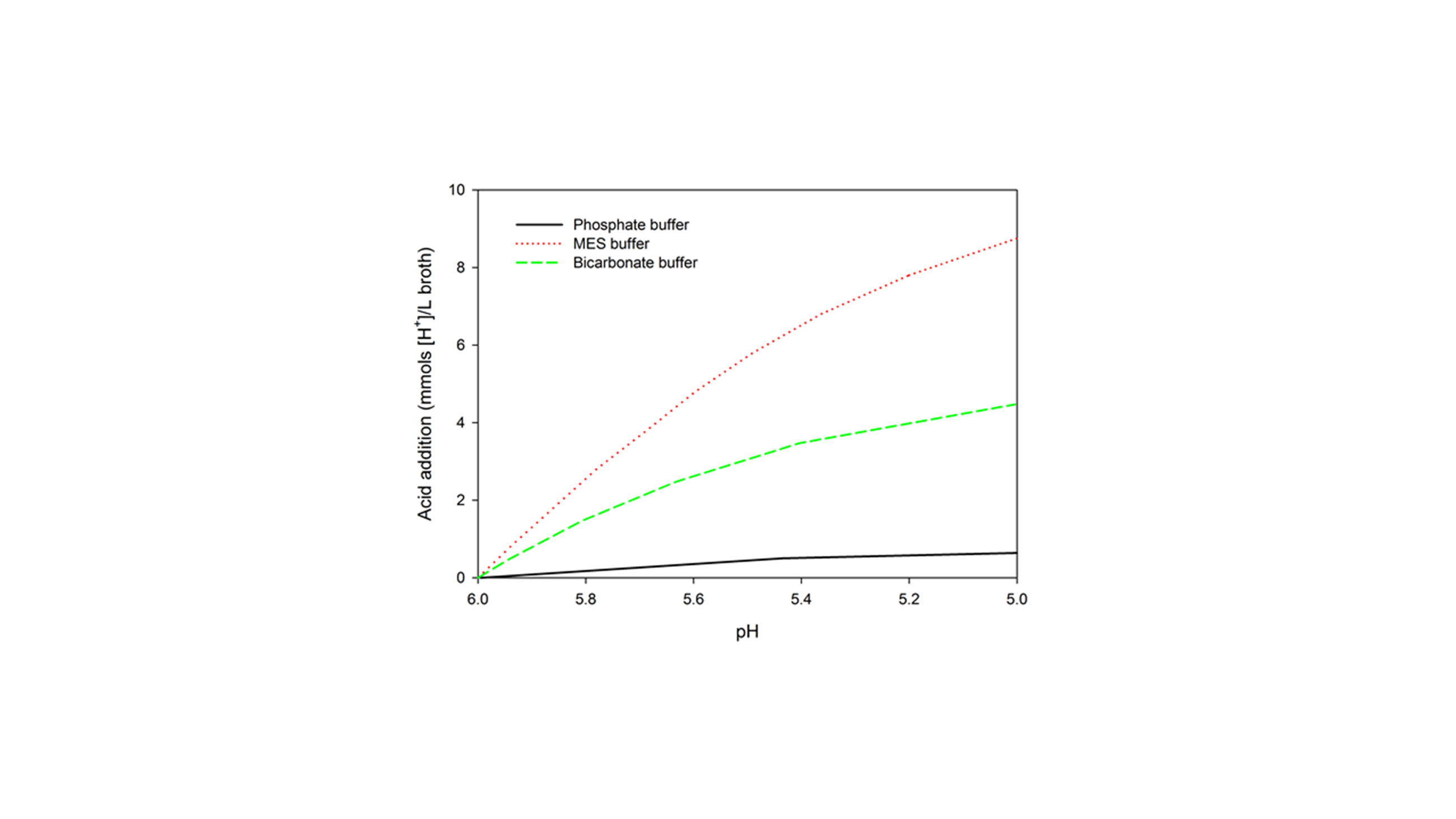

Supplement: Supplementary file 6 [file Image6.TIF]

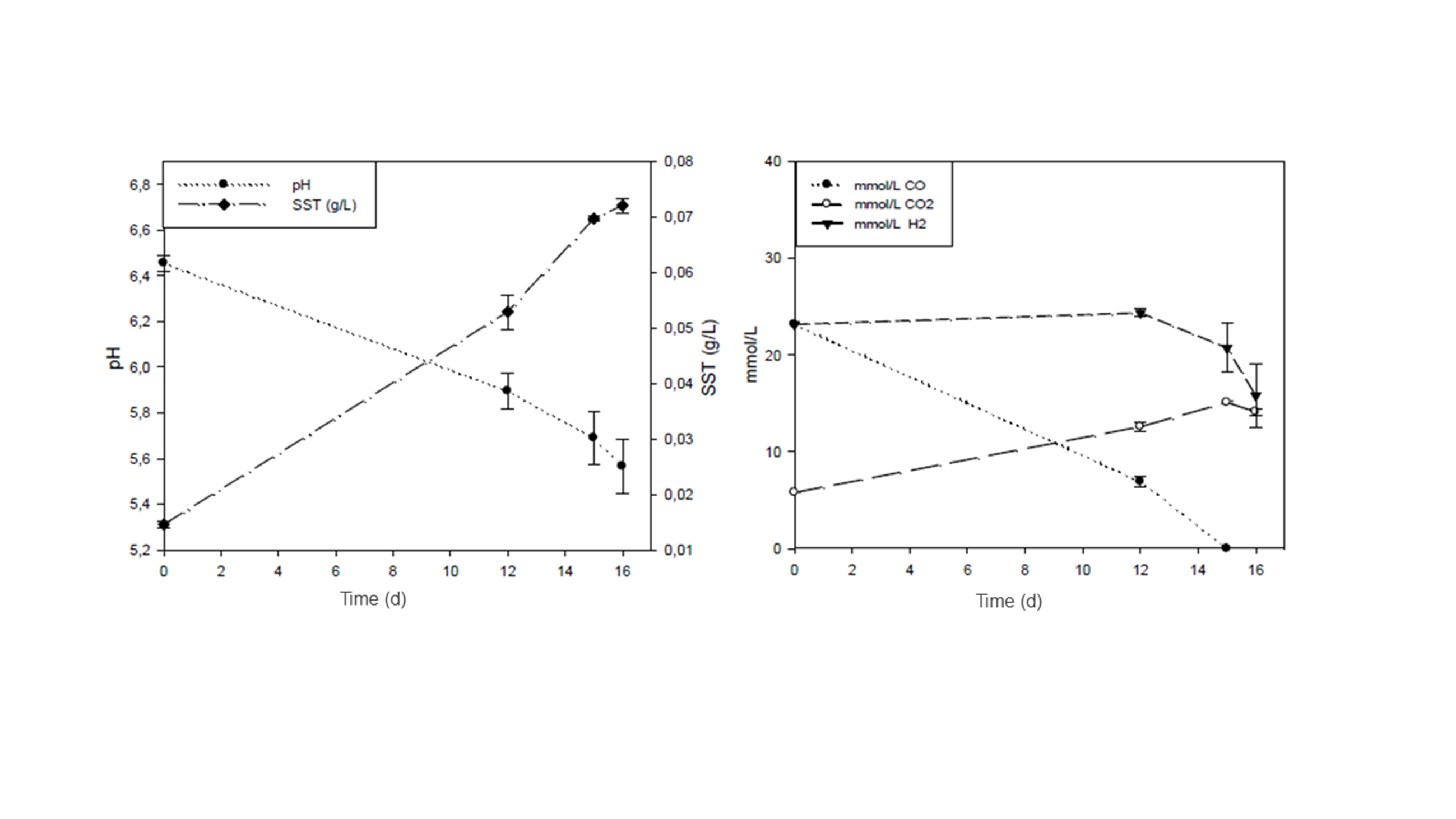

Supplement: Supplementary file 7 [file Image7.TIF]
